# Clinical Overestimation of HER2 Positivity in Early Estrogen and Progesterone Receptor–Positive Breast Cancer and the Value of Molecular Subtyping Using BluePrint

**DOI:** 10.1200/JGO.2016.006072

**Published:** 2016-11-16

**Authors:** Ettienne J. Myburgh, Lizanne Langenhoven, Kathleen A. Grant, Lize van der Merwe, Maritha J. Kotze

**Affiliations:** **Ettienne J. Myburgh** University of Stellenbosch, Tygerberg; **Lizanne Langenhoven**, Mediclinic Panorama Hospital, Cape Town; **Kathleen A. Grant**, Cape Peninsula University of Technology; **Lize van der Merwe**, University of Western Cape, Bellville; and **Maritha J. Kotze**, University of Stellenbosch and National Health Laboratory Service, Tygerberg Hospital, Cape Town, South Africa.

## Abstract

**Purpose:**

Human epidermal growth factor receptor 2 (HER2) positivity is an important prognostic and predictive indicator in breast cancer. HER2 status is determined by immunohistochemistry and fluorescent in situ hybridization (FISH), which are potentially inaccurate techniques as a result of several technical factors, polysomy of chromosome 17, and amplification or overexpression of CEP17 (centromeric probe for chromosome 17) and/or HER2. In South Africa, HER2-positive tumors are excluded from a MammaPrint (MP; Agendia BV, Amsterdam, Netherlands) pretest algorithm. Clinical HER2 status has been reported to correlate poorly with molecular subtype. The aim of this study was to investigate the correlation of clinical HER2 status with BluePrint (BP) molecular subtyping.

**Methods:**

Clinico-pathologic and genomic information was extracted from a prospectively collected central MP database containing records of 256 estrogen receptor–positive and/or progesterone receptor–positive tumors. Twenty-one tumors considered HER2 positive on immunohistochemistry or FISH were identified for this study.

**Results:**

The median age of patients was 56 years (range, 34 to 77 years), with a median tumor size of 16 mm (3 to 27 mm). Four (19%) tumors were confirmed HER2-enriched subtype, six (29%) were luminal A, and 11 (52%) were luminal B. The positive predictive values of HER2/CEP17 ratio ≥ 2 and HER2 copy number ≥ 6 were only 29% and 40%, respectively. The differences in means for HER2/CEP17 ratio were significant between BP HER2-enriched versus luminal (*P* = .0249; 95% CI, 0.12 to 1.21) and MP high-risk versus low-risk tumors (*P* = .0002; 95% CI, 0.40 to 1.06).

**Conclusion:**

Of the 21 tumors considered clinically HER2 positive, only four were HER2-enriched subtype with BP, indicating an overestimation of HER2 positivity. FISH testing has a poor positive predictive value.

## INTRODUCTION

Human epidermal growth factor receptor 2 (*HER2*), or *HER2/neu*, has been a well-recognized oncogene involved in the oncogenesis of several cancers. In breast cancer, it is an established prognostic and predictive biomarker.^1^ Where the adjuvant management of early HER2-positive breast cancer is concerned, significant improvements in disease-free and overall survival have been shown with the addition of trastuzumab to chemotherapy for tumors > 1 cm and for node-positive disease.^2-6^ This benefit was also seen for tumors < 1 cm when certain adverse prognostic features were present.^7^

Screening mammography has given rise to an increased incidence of breast cancer diagnosed at an earlier stage,^8^ of which an estimated 15% to 20% are considered to be HER2 positive.^1^ In southern Africa, the incidence of HER2-positive breast cancer is estimated at 25.2%.^9^ Although data before 2011 were limited by under-reporting from private laboratories, the effect of this was found to be < 4%.^10^ Because of the cost implications of trastuzumab on a developing economy, only a minority of these patients have access to trastuzumab at any stage of their disease. Significant disparity exists between patients who rely on state care and those who have access to private medical insurance.

For patients who rely on state care, trastuzumab is only available in one of the nine South African provinces. Insofar as private funders are concerned, reimbursement protocols are diverse and often require a significant copayment from the patient. It is not uncommon to administer trastuzumab only to a certain arbitrary financial point rather than according to standard of care guidelines, which currently suggest 1 year of adjuvant therapy. In such a cost-conscious environment, all attempts should be made to more clearly define a subset of patients who may benefit from costly therapies, thereby improving access by all women regardless of financial or social means.

Identifying patients with HER2-positive tumors has relied largely on immunohistochemistry (IHC) and the use of fluorescent in situ hybridization (FISH). Unfortunately, several pitfalls exist in the process. Notably, these include the variability in reporting of HER2 status by IHC assessment only, and the effect of polysomy of chromosome 17 in breast tumor cells leading to inaccurate estimation of *HER2* gene expression.^11^ Guidelines have been developed to improve the accuracy of reporting, with the intent of selecting any patient who might possibly benefit from targeted therapy.^12^ This has led to more equivocal cases being identified.^13^ In a cost-conscious environment, however, we should focus our attention on modalities that might maintain the positive predictive value (PPV) but also improve the negative predictive value to avoid ineffective and costly therapy.

In South Africa and Namibia, most health care funders require reflex FISH testing on all HER2-positive tumors with 2+ or 3+ IHC staining, although some funders will accept tumors with 3+ IHC staining for the funding of trastuzumab.

Since 2007, it has been possible to order MammaPrint (MP; Agendia BV, Amsterdam, Netherlands) through local agents in South Africa, which shipped the prepared material to the Netherlands. MP is a 70-gene microarray-based genomic risk–assessment assay. In 2010, an 80-gene microarray called BluePrint (BP) was added to report the intrinsic molecular subtype of the tumor. The HER2-enriched subtype is defined by the *ERBB2*, *GRB7*, *PERLD1*, and *SYCP3* genes.^14^ Until early 2016, the Agendia Breast Cancer Suite also included a separate single-gene microarray for the estrogen receptor (ER), progesterone receptor (PR), and HER2 receptor, namely TargetPrint (TP).

After an initial health economic assessment and the development of an MP pretest algorithm (MPA),^15^ this technology was introduced for early-stage ER/PR-positive, HER2-negative tumors in South Africa; to date, 256 tests have been done. In a recent publication, Pohl et al^16^ showed that MP altered the treatment decision in 52% of patients in this subgroup. With the recent publication of the results from the EORTC-10041/BIG-3-04 MINDACT (Microarray In Node-Negative and 1 to 3 Positive Lymph Node Disease May Avoid Chemotherapy) trial, the safety of omitting adjuvant chemotherapy in low-risk tumors, as determined by MP, has been well established.^17^ As in the MINDACT trial, which included 9.5% HER2-positive tumors, a few such tumors were included in the South African MP database.

Internationally, several other genomic profiling assays have become available, notably Oncotype DX, which is the only other assay available to the South African private health sector through local agents. Although it has approximately 90% of the market share in the United States,^18^ it is estimated to hold roughly 40% of the market share in South Africa. It does not report molecular subtyping.

Although recently published guidelines from ASCO recommended against the use of any commercially available biomarkers in early HER2-positive breast cancer,^19^ there has been substantial evidence of the importance of molecular subtyping in treatment decision making rather than pure recurrence risk prediction,^20,21^ which would be more appropriate in the era where targeted biologic agents are being developed at a rapid pace.

The negative predictive value of single-gene HER2 reporting has been shown in a cohort of patients with HER2-negative tumors.^22^ In HER2-positive tumors, mRNA levels seem to be a good predictor of response to trastuzumab and chemotherapy in patients with ER-positive disease.^23^

It is well recognized that current strategies in HER2 testing do not accurately determine all of the molecular pathways driving oncogenesis. This might be the reason why a complete pathologic response was only seen in 31.7% of patients in the HER2-positive subset of the GeparQuattro data set.^24^ Several publications have recently alluded to the fact that a significant proportion of tumors reported to be HER2 positive using IHC/FISH (clinically HER2 positive [cHER2]) are not of the HER2-enriched (HER2E) subtype.^19,25,26^ This indicates that other pathways might drive proliferation, eg, downstream phosphatidylinositol 3-kinase activation through mechanisms such as loss of PTEN, which induces resistance to trastuzumab.^27^ In a publication by Prat et al,^26^ only 47% of patients with cHER2 tumors had the HER2E subtype. In the Neoadjuvant Breast Registry Symphony Trial, only 33 (44%) of the 75 patients with ER/PR-positive cHER2-positive disease had the HER2E subtype.^20^

To further investigate the role of genomic profiling beyond standard HER2 reporting, this study was undertaken to evaluate the value of molecular subtyping in HER2 assessment of ER/PR-positive breast cancers.

## METHODS

This study was performed in accordance with the ethical standards laid out in the 1964 Declaration of Helsinki. Ethical approval was granted by the Health and Research Ethics Committee of the University of Stellenbosch (reference number N09/06/166). All patients consented to their clinico-pathologic data being stored in a central database and used for later analysis.

### Study Population

Detailed records of 256 tumors from southern African patients, referred for MP analysis since 2007, have been prospectively collected in a central database. Referrals were from oncologists and surgeons in both private and public practices in South Africa and Namibia. Histology and FISH testing was done by several nationally accredited private pathology laboratories, and no central review was done. MP risk scoring and ER, PR, and HER2 receptor status were determined as previously described.^22^ Records of the tumors that had BP assays performed were considered and provided information on patient age and sex; tumor type, grade, and size; ER, PR, and HER2 status; nodal status; MP risk; and the molecular subtype as determined by BP. Most also had TP results for ER, PR, and HER2.

### Inclusion Criteria

Patients with equivocal or positive HER2 testing as defined by the 2013 American Society of Clinical Oncology/College of American Pathologists consensus guidelines,^12^ including HER2 staining intensity of 3+ (IHC), HER2/centromeric probe for chromosome 17 (CEP17) ratio ≥ 2 (FISH), or HER2 copy number ≥ 4 (FISH).

### Statistical Analysis

Characteristics are described as the median (range) for quantitative/numerical data and as count (percentage) for qualitative/categorical data. Because the variances of the groups in both the MP and the BP result groups were clearly different, Welch two-sample *t* tests were used to compare the mean values of both HER2/CEP17 ratio and HER2 copy number between each of the BP and MP results. To ensure valid results, both HER2/CEP17 ratio and HER2 copy number were log-transformed to approximate normality before analysis, because of data skewness. Diagnostic measures were estimated from scratch. Functions from R, freely available from www.r-project.org, were used for the statistical analysis.

## RESULTS

From the database, 21 tumors were identified by our selection criteria (Table 1). The demographic data of the study population are summarized in Table 2. Patients were all women, with a median age of 56 years (range, 34 to 77 years), and with histologically confirmed infiltrating ductal carcinomas, which were moderately to strongly ER positive. Seven of the tumors were PR negative. The histologic grades of differentiation were predominantly grade 2 (10 tumors, 47%) and grade 3 (eight tumors, 38%); two tumors were grade 1, and one was of unknown differentiation. The median tumor size was 16 mm (range, 3 to 27 mm). Six (29%) patients with nodal involvement were included, five of whom had macrometastases to one node, and one patient with isolated tumor cells only.

**Table 1:**
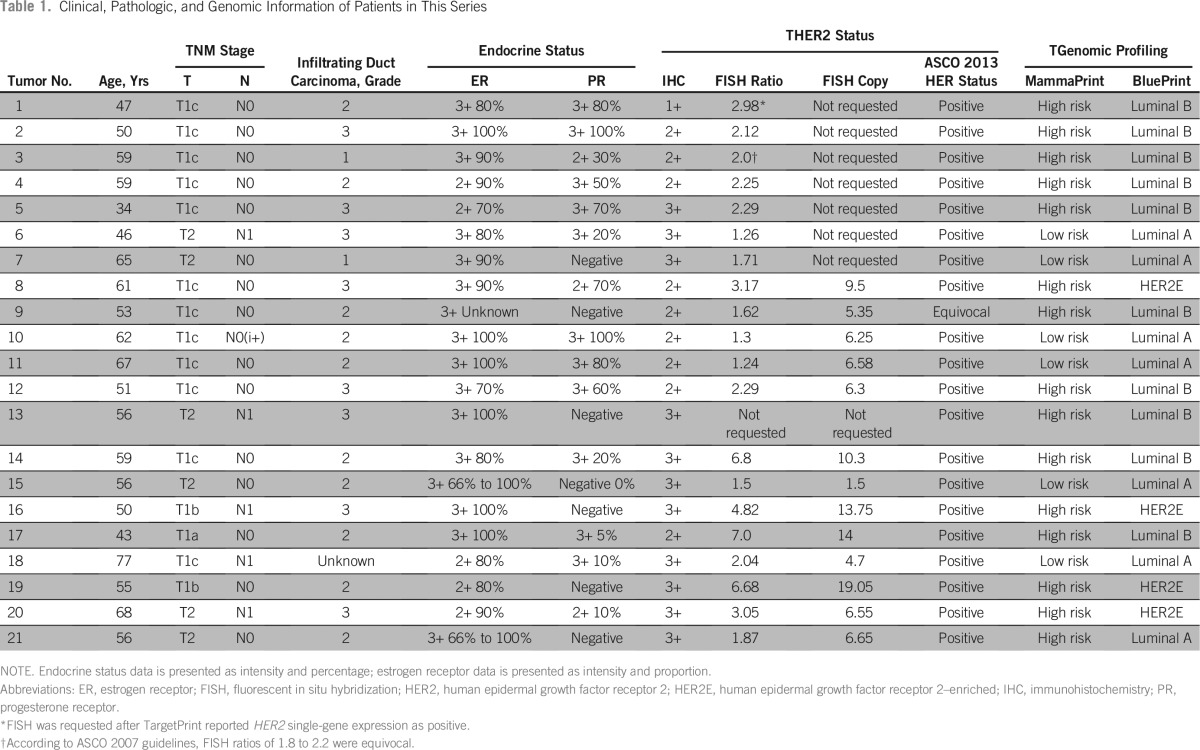
Clinical, Pathologic, and Genomic Information of Patients in This Series

**Table 2:**
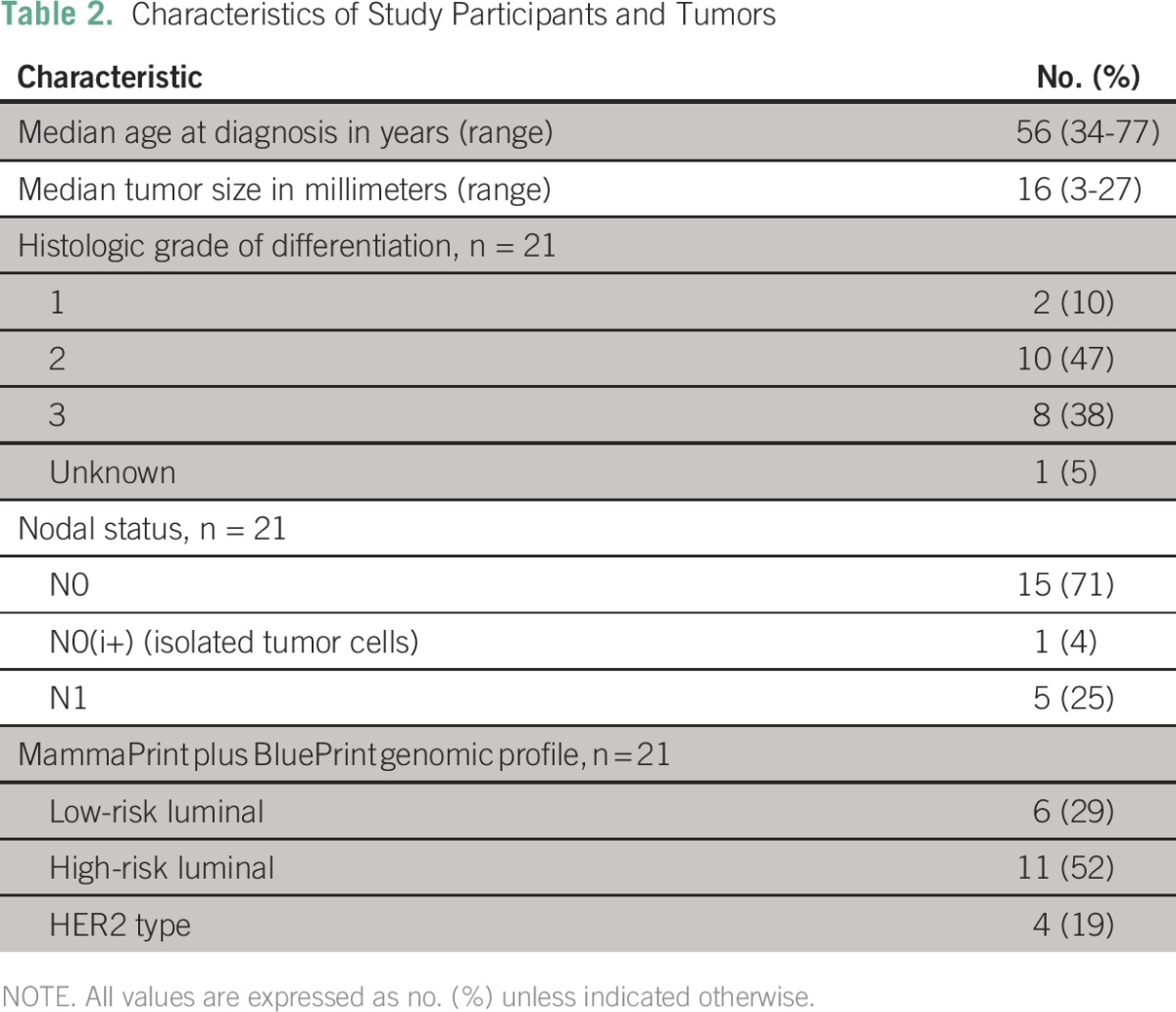
Characteristics of Study Participants and Tumors

FISH tests were done in 20 patients, among whom seven (35%) tests reported only the HER2/CEP17 ratio because they were done before 2013.

Eleven (52%) tumors had IHC HER2 staining intensity of 3+, of which seven (64%) were positive on FISH testing, three (27%) were negative, and one was not sent for FISH analysis. Nine (42%) patients with HER2 staining intensity of 2+ included eight (89%) who were considered HER2 positive and one equivocal on FISH applying the ASCO 2013 criteria.^12^ Of the eight positives, six (75%) showed a HER2/CEP17 ratio ≥ 2 and two (25%) had ratios < 2 but copy number > 6. One (5%) tumor remained equivocal after FISH. One (5%) patient, with HER2 staining intensity of 1+ and luminal subtype, had overexpression of HER2 mRNA on TP and subsequently showed an HER2/CEP17 ratio of 2.98 on reflex FISH testing.

Of note, referring physicians requested reflex FISH analyses for another five of the patients. In one patient, the initial HER2/CEP17 ratio was equivocal based on ASCO 2007 guidelines,^27a^ and the same result was found after reflex FISH. In four patients, FISH was repeated after BP reported luminal subtype and in three of them, the HER2/CEP17 ratios were discordant and in the fourth, the copy number was discordant (Table 3).

**Table 3:**
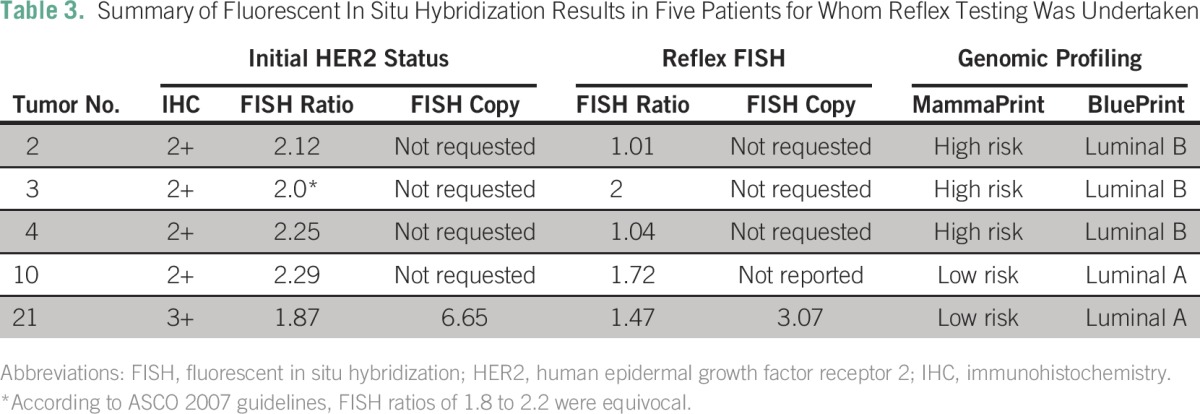
Summary of Fluorescent In Situ Hybridization Results in Five Patients for Whom Reflex Testing Was Undertaken

On the basis of the ASCO 2013 guidelines,^12^ 20 (95%) patients would have been considered HER2 positive and one inconclusive, requiring further testing.

**Fig 1:**
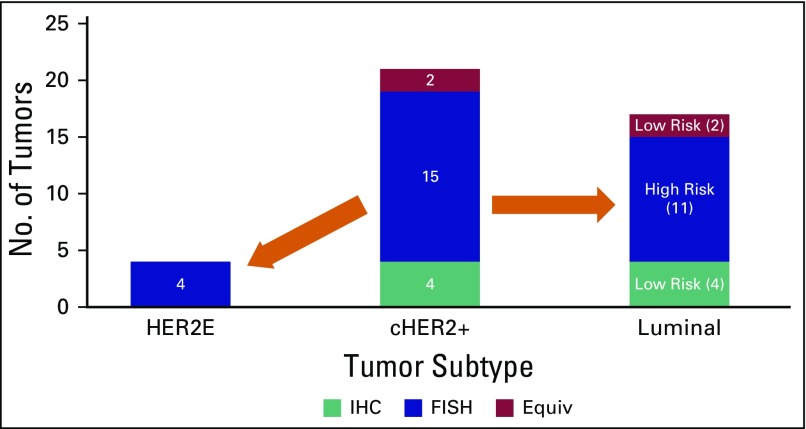
Reclassification of tumors reported to be clinically positive for human epidermal growth factor receptor 2 (cHER2+) using immunohistochemistry (IHC)/fluorescent in situ hybridization (FISH) into HER2-enriched (HER2E) and luminal subtypes. Equiv, equivocal.

On BP analysis with MP risk, only four (19%) tumors were HER2E subtype, six (29%) were low-risk luminal, and 11 (52%) were high-risk luminal (Fig 1).

**Fig 2:**
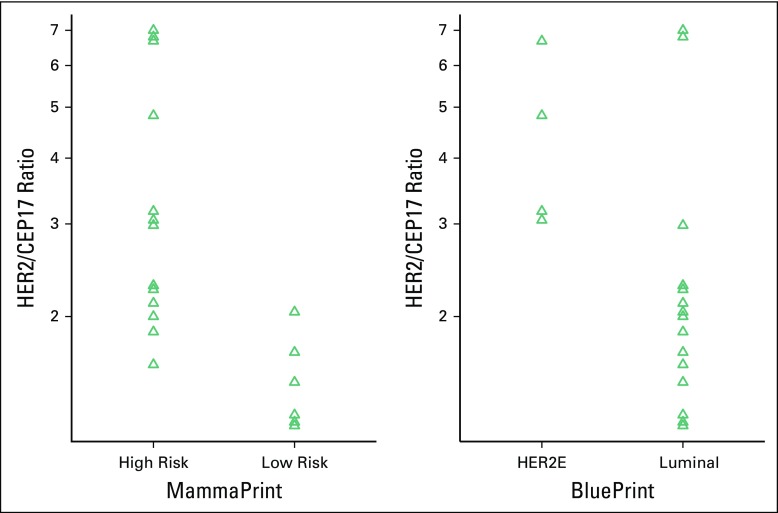
Strip plots of human epidermal growth factor receptor 2 (HER2)/centromeric probe for chromosome 17 (CEP17) ratios on log-scale for both MammaPrint risk and BluePrint subtyping. Distributions are closer to normality, but variances remain different. The difference in means of the HER2/CEP17 ratio was significant for molecular subtype (*P* = .0249; 95% CI, 0.12 to 1.21 logs) and MammaPrint risk (*P* = .0002; 95% CI, 0.40 to 1.06 logs).

Assuming that BP accurately identifies HER2E subtype and taking into account the initial FISH analysis in 20 patients, as well as the results of five reflex tests (n = 25), an HER2/CEP17 ratio ≥ 2 had a sensitivity of 100% (four of four), but a PPV of only 29% (four of 14) and a Matthews correlation coefficient of 0.387. Similarly, in 14 FISH tests where the HER2 copy number was reported, a value ≥ 6 had a sensitivity of 100% (four of four), but a PPV of only 40% (four of 10) and a Matthews correlation coefficient of 0.40. The difference in means of the HER2/CEP17 ratio was significant for HER2E versus luminal tumors (*P* = .0249; 95% CI for true difference in log-transformed means, 0.12 to 1.21 logs; Fig 2).

Using the 2013 ASCO guidelines to predict HER2 positivity and excluding one equivocal result, the PPV was only 20% (four of 20). Interestingly, the difference in means of the HER2/CEP17 ratio was highly significant for MP high-risk versus low-risk tumors (*P* = .0002; 95% CI for true difference in log-transformed means, 0.40 to 1.06). In this instance, a ratio > 2 has a specificity of 92%, a PPV of 0.85, and a negative predictive value of 0.83 for a high-risk MP result.

## DISCUSSION

The current series is limited by small numbers as a result of the exclusion of cHER2-positive patients from MP funding in southern Africa, in accordance with the MPA initially described by Grant et al.^15^ Valuable information could nevertheless be gleaned from the 21 HER2-positive tumors extracted from the MP database, which indicated a possible overestimation of HER2 status. Only four of the patients had HER2E tumors, suggesting a good response to trastuzumab treatment. The majority, however, had luminal A or B disease, with a significantly lower reported response to chemotherapy and doubtful benefit from trastuzumab.^19,24,25^ The six low-risk luminal A tumors would be expected to respond well to endocrine therapy only.

Published data from South Africa on 109 ER/PR-positive, HER2-negative tumors showed a high risk-to-low risk ratio of 39% versus 61%,^16^ whereas 68% of patients in the current series were high risk on MP, indicating that FISH positivity might predict a higher likelihood of a high-risk result but has poor PPV for HER2E.

If results from the Neoadjuvant Breast Registry Symphony Trial^20^ are considered, a pathologic complete response (pCR) was seen in 53% of HER2E subtypes versus 38% in cHER2-positive patients. In IHC/FISH ER/PR-positive, HER2-positive patients, reclassified as luminal A or B using BP, the pCR was only 3%. The difference in pCR in cHER2 versus HER2E tumors can be explained by the dilutional effect of the large proportion of patients with luminal disease in the cHER2 group. If these results were applied to the current cohort, the expected benefit of trastuzumab would be even less, considering the large percentage of patients with luminal breast cancer. Furthermore, it seems that omitting anti-HER2 therapy in patients with non-HER2E tumors does not have an impact on survival.^26^

From our data analysis, it was clear that the current ASCO guidelines for HER2/CEP17 ratio and copy number has a poor PPV. It is possible that using higher cut-off values might reduce the number of luminal tumors selected; some authors have reported a better response to trastuzumab with copy number > 12^28^ and higher HER2/CEP17 ratio,^29^ although the HER2E tumors in this series have copy numbers ranging from 6.55 to 19.05 and HER2/CEP17 ratios from 3.17 to 6.68.

A significant difference in the log-transformed mean HER2/CEP17 ratio and copy number was observed for HER2E tumors compared with the luminal tumors. The small numbers and skewness resulted in wide confidence intervals with significant overlap, which precluded further analysis of the series. Because of this, reporting the actual mean values will not be a reflection of the true center of the data set. It is, however, a phenomenon that could be explored in a future larger series.

The fact that the four patients had contradictory HER2/CEP17 ratios or copy number on repeat testing is a further indication of the discrepancies in the current testing practice. Despite the small data set and wide confidence intervals, the PPV for both HER2/CEP17 ratio and copy number remains poor.

Two node-negative tumors in this series were < 10 mm; one was found to be high-risk luminal and one HER2E. In these small tumors, the importance of accurate HER2 determination is of even greater consequence. It is generally accepted that small node-negative tumors have a good prognosis, but there remains a small number of patients with poor outcomes. Little prospective information is available in T1a/b tumors, and only the Breast Cancer International Research Group 006 trial (BCIRG-006) allowed inclusion of node-negative tumors < 10 mm.^6^ However, with reported 5-year distant recurrence–free survival of 98% in untreated HER2-positive T1a/b tumors,^30^ the benefit from adjuvant chemotherapy and trastuzumab is possibly outweighed by the toxicity of treatment^31^ in this subgroup of patients. Even with alternative chemotherapy regimens, toxicity remains high, with limited benefit in patients with tumors < 10 mm.^32^ It might therefore be helpful to submit all node-negative cHER2 tumors < 10 mm for MP/BP testing to select those patients most likely to gain benefit.

The numbers and demographic characteristics of the study population are limited by the referral bias imposed by the pre-existing MPA^15^ and conservative selection of patients by referring physicians. This is reflected in the small percentage of HER2-positive tumors contained in the database. Furthermore, the study focused on a subgroup of ER/PR-positive tumors only. In most of the published series evaluating HER2-positive tumors, both hormone receptor–positive and hormone receptor–negative tumors were included, and a difference in response to trastuzumab between these two groups was reported.^19^ Additionally, the predictive value of MP/BP needs further validation in selecting patients for trastuzumab treatment.

Despite the limitations of this study, it is in line with similar reports of larger series. As a preliminary investigation, it warrants the development of expanded referral guidelines and further evaluation of molecular subtyping in patients with cHER2-positive tumors.

On the basis of our findings in this study and the recent evidence from literature of the overestimation of HER2 positivity as determined by clinical guidelines, further evaluation of molecular subtyping in the work up of HER2-positive tumors should be considered,^33^ including ER/PR-negative, cHER2-positive tumors < 10 mm. Institutional protocols, such as the MPA applied as a cost-saving strategy in the southern African context,^15,16^ should be adapted to include patients with ER/PR-positive, cHER2-positive disease with limited nodal involvement to match the demographic characteristics of those reported in the MINDACT trial.
